# Transferable antibiotic resistance plasmids from biogas plant digestates often belong to the IncP-1ε subgroup

**DOI:** 10.3389/fmicb.2014.00765

**Published:** 2015-01-21

**Authors:** Birgit Wolters, Martina Kyselková, Ellen Krögerrecklenfort, Robert Kreuzig, Kornelia Smalla

**Affiliations:** ^1^Julius Kühn-Institut, Federal Research Centre for Cultivated Plants (JKI), Institute for Epidemiology and Pathogen DiagnosticsBraunschweig, Germany; ^2^Technische Universität Braunschweig, Institute of Environmental and Sustainable ChemistryBraunschweig, Germany; ^3^Biology Centre of the Academy of Sciences of the Czech Republic, Institute of Soil BiologyČeské Budějovice, Czech Republic

**Keywords:** IncP-1ε plasmid, class 1 integrons, biogas plant digestate, antibiotic resistance, exogenous plasmid isolation

## Abstract

Manure is known to contain residues of antibiotics administered to farm animals as well as bacteria carrying antibiotic resistance genes (ARGs). These genes are often located on mobile genetic elements. In biogas plants (BGPs), organic substrates such as manure and plant material are mixed and fermented in order to provide energy, and resulting digestates are used for soil fertilization. The fate of plasmid carrying bacteria from manure during the fermentation process is unknown. The present study focused on transferable antibiotic resistance plasmids from digestates of seven BGPs, using manure as a co-substrate, and their phenotypic and genotypic characterization. Plasmids conferring resistance to either tetracycline or sulfadiazine were captured by means of exogenous plasmid isolation from digestates into *Pseudomonas putida* KT2442 and *Escherichia coli* CV601 recipients, at transfer frequencies ranging from 10^-5^ to 10^-7^. Transconjugants (*n* = 101) were screened by PCR-Southern blot hybridization and real-time PCR for the presence of IncP-1, IncP-1ε, IncW, IncN, IncP-7, IncP-9, LowGC, and IncQ plasmids. While 61 plasmids remained unassigned, 40 plasmids belonged to the IncP-1ε subgroup. All these IncP-1ε plasmids were shown to harbor the genes *tet*(A), *sul1*, *qacEΔ1, intI1,* and integron gene cassette amplicons of different size. Further analysis of 16 representative IncP-1ε plasmids showed that they conferred six different multiple antibiotic resistance patterns and their diversity seemed to be driven by the gene cassette arrays. IncP-1ε plasmids displaying similar restriction and antibiotic resistance patterns were captured from different BGPs, suggesting that they may be typical of this environment. Our study showed that BGP digestates are a potential source of transferable antibiotic resistance plasmids, and in particular the broad host range IncP-1ε plasmids might contribute to the spread of ARGs when digestates are used as fertilizer.

## INTRODUCTION

Due to the promotion of renewable energies the number of biogas plants (BGPs) in Germany is on the rise during the last years (Fachverband Biogas e.V., 2014^1^). In BGPs organic substrates of different origin, such as manures, sludge or plant material, are mixed and fermented anaerobically under usually mesophilic conditions (approximately 40°C) in order to provide energy (Weiland, 2010). The conversion of biomass into biogas is accomplished via enzymatic degradation driven by bacteria and archaea. The digestates resulting from this process are often stored for several months before being applied to agricultural fields as fertilizer similar to manures.

Depending on their chemical structure, antibiotics applied to livestock, as well as their partially still active metabolites, were detected in the excrements of treated animals in several studies (Boxall et al., 2004; Sarmah et al., 2006; Lamshöft et al., 2007). Thus, antibiotics present in manure might select for resistant bacteria. Consistently, in multiple studies piggery manure was reported as a reservoir of bacteria carrying antibiotic resistance genes (ARGs; Hölzel et al., 2009) and genes conferring resistance to all major classes of antibiotics have been detected in total community (TC-) DNA of pig manures and slurries (Marti et al., 2013; Zhu et al., 2013). In addition, ARGs from manure bacteria were shown to be often located on mobile genetic elements (MGEs) such as plasmids (Binh et al., 2008; Heuer et al., 2012) and transferable plasmids of several different incompatibility groups, e.g., IncP-1, IncN, IncW, IncP-9, LowGC, and IncQ plasmids (Krasowiak et al., 2002; Binh et al., 2008; Heuer et al., 2009), have been reported frequently in TC-DNA from pig manures and slurries. Some of these plasmid groups, such as IncP-1, IncN, IncW, and IncQ have a broad host range and may be exchanged among phylogenetically distant bacteria (Pukall et al., 1996; Shintani et al., 2010, 2014; Suzuki et al., 2010; Klümper et al., 2014). Therefore, when spreading manure on field soils, ARGs located on MGEs might be transferred to soil bacteria. Indeed, the IncP-1α plasmid pGP527 and the mobilizable IncQ plasmid pIE723 were shown to be transferred to *Pseudomonas putida* under field conditions when introduced together with manure (Götz and Smalla, 1997). Thus, the use of manure as fertilizer might contribute to the spread of antibiotic resistance in agricultural settings (Rahube and Yost, 2012; Jechalke et al., 2014b).

Another group of genetic elements assumed to play an important role in the dissemination of ARGs are integrons that are often associated with the plasmid groups mentioned above (Thorsted et al., 1998; Bahl et al., 2007; Eikmeyer et al., 2012; Heuer et al., 2012). Although not mobile *per se*, integrons are often linked to MGEs, such as plasmids, IS*CR* (insertion sequence common region) elements or transposons (Mazel, 2006), but may also be located on chromosomes (Mazel et al., 1998; Boucher et al., 2007). Due to their ability to capture, integrate, express and excise promoter-less gene cassettes, which in many cases code for ARGs, integrons function as a genetic platform for the exchange and accumulation of antibiotic resistance determinants in host bacteria (Stokes and Hall, 1989; Boucher et al., 2007). Thereby they allow for a fast adaptation of their host to antibiotic stress by integration of new gene cassettes, and also further extend the accessibility of ARGs to bacteria when being located on transferable plasmids or other MGEs. It has been shown that integrons contribute to the spread of ARGs in the environment as well as in clinical settings (Gillings, 2014). They are widely distributed in Gram-negative bacteria (including pathogens) and are also present in Gram-positive bacteria (Moura et al., 2009; Gillings, 2014).

Land application of animal manure has been recognized as a risk factor for the dissemination of MGEs associated with ARGs in soil (*reviewed in* e.g., Chee-Sanford et al., 2009; Heuer et al., 2011), but whether digestates from BGPs that use manure as a co-substrate represent a similar risk remains to be evaluated. So far, microbiological research on BGPs has focused mainly on the fermenter, while less attention has been paid to the stored digestates. Nevertheless, several studies analyzed the influence of the biogas process on the survival of pathogenic or multiresistant bacteria (Sahlström, 2003; Iwasaki et al., 2011; Beneragama et al., 2013; Resende et al., 2014). For example, Beneragama et al. (2013) showed at the lab scale that digestates which had been treated at mesophilic temperatures still contained viable multidrug resistant bacteria originating from the fermented manure. But yet the question of whether such bacteria contain transferable ARGs that might be transferred to bacteria associated with soil or plants remained unanswered.

The present study thus aimed to explore whether digestates of mesophilic full-scale BGPs fed with manures represent a possible source of transferable antibiotic resistance plasmids and to characterize these plasmids in terms of genotypic (incompatibility groups, presence of ARG and integrons, restriction patterns) and phenotypic (conferred antibiotic resistance) diversity.

## MATERIAL AND METHODS

### SAMPLING

Stored digestates from seven BGPs were sampled (four replicates per BGP) in Lower Saxony during autumn 2012. Six of these BGPs were fermenting pig manure and one alternatively cattle manure as co-substrate in combination with varying plant material. Storage tanks were filled semi-continuously during the year with freshly digested material from the fermenters or postfermenters of the BGPs and mixed. Sampling was performed from storage tanks after thoroughly stirring the digestates and was done either via tank outlet valves or, in case of open silos, via probe samplers, bypass samplers or in back-flush mode from a vacuum tanker. For further information on fermented materials and other features of the different BGPs, see **Table 1**.

**Table 1:** Substrates used in sampled biogas plants (BGPs), resilience time of material within fermenters, storage system and time periods of semi-continuous digestate collection (storage time).

BGP#	Substrates	Residence time in fermenter [d]	Storage of digestates	Storage time digestates [months]
1	Pig manure, maize silage, potato mash, few beet	100	Open	(No information)
2	Pig manure, maize silage, gras, sunflower silage, onions, cattle manure	100	Closed	(No information)
3	Pig manure, maize silage	90–100	Closed	8–9
4	Pig manure, maize silage	90–100	Storage in postfermenter	8–9
5	Pig manure, maize silage, dry chicken manure	67	Closed	2
6	Pig manure, bull manure, dry chicken manure, maize, green rye	(No information)	Closed	(No information)
7	Cattle manure, maize silage, feed rests, dung	(No information)	Closed	(No information)

### SAMPLE PREPARATION

For exogenous plasmid isolation aliquots of digestate sample replicates were pooled and processed shortly after sampling. Fifty mL of the resulting homogenized samples were centrifuged in 50 mL Falcon tubes at 3,100 ×*g* for 10 min at room temperature (RT). Pellets were resuspended and washed twice in 30 mL sterile 0.85% NaCl solution followed by centrifugation for 2 min at 3,100 ×*g* at RT. Supernatants were discarded, resulting pellets were homogenized manually with sterile spatula and used immediately as donors for exogenous plasmid isolation.

### STRAINS

*Escherichia coli* CV601 *gfp*^+^ [resistant toward kanamycin (KAN) and rifampicin (RIF), Heuer et al., 2002] and *P. putida* KT2442 *gfp*^+^ (resistant toward KAN, RIF, Heuer et al., 2002) were used as recipient strains in exogenous plasmid isolation. *E. coli* DH5α (no antibiotic resistance) was used as recipient for subsequent transformation of captured plasmids via electroporation.

### EXOGENOUS PLASMID ISOLATION

Single colonies of recipient strains were inoculated into 40 mL sterile Luria Bertani broth (LB; Roth, Karlsruhe, Germany) supplemented with KAN (50 mg/L) and RIF (50 mg/L) in 100 mL Erlenmeyer flasks and incubated for 20 h at 28°C and 150 rpm on a shaker. One mL of each recipient culture was centrifuged at 3,100 ×*g* for 5 min at RT. Pellets were washed twice with 1 mL 1/10 tryptic soy broth (TSB; Merck, Darmstadt, Germany), centrifuged at the same conditions and dispersed in 1 mL 1/10 TSB. Five g of each digestate pellet (see sample preparation) were dissolved in 45 mL sterile 1/10 TSB in 100 mL Erlenmeyer flasks and pre-incubated at 28°C and 150 rpm for 2 h on a shaker. 1,950 μL of each digestate suspension were mixed with 50 μL of recipient suspension in a 2 mL reaction tube in order to prepare mating mixes. 1,950 μL of digestate suspension without recipient and 50 μL of recipient suspension added to 1,950 μL of sterile 1/10 TSB were used as donor and recipient controls, respectively. The tubes were centrifuged at 16,000 ×*g* for 5 min at RT (Centrifuge 5402, Eppendorf, Hamburg, Germany), supernatants were removed carefully with a pipette and resulting pellets were resuspended in 100 μL 1/10 TSB. The suspensions were transferred to Millipore filters (0.22 mm) placed on plate count agar (PCA; Merck) supplemented with cycloheximide (100 mg/L) and incubated overnight at 28°C. Afterward the filters were transferred into 50 mL Falcon tubes containing 10 mL sterile 0.85% NaCl solution and vortexed for 1 min to detach the cells from the filters (corresponding to a 10^-2^ dilution). Serial dilutions (10^-2^ to 10^-9^) were prepared from the cell suspensions with sterile 0.85% NaCl solution. To obtain transconjugants that captured resistance toward sulfonamides, 100 μL of serial dilutions (10^-2^ to 10^-4^) of *E. coli* CV601 *gfp*^+^ mating mixes were plated in duplicate on Mueller-Hinton agar according to CLSI (Merck; this medium is recommended for sulfonamide testing) supplemented with KAN (50 mg/L), RIF (50 mg/L) and sulfadiazine (SDZ; 50 mg/L). Alternatively, for isolation of *P. putida* KT2442 *gfp*^+^ transconjugants that captured tetracycline (TET)-resistance, 100 μL of serial dilutions (10^-2^ to 10^-4^) were streaked in duplicate on R2A agar (Merck; this medium prevents excessive exopolysaccharide production by *P. putida* which might disturb subsequent plasmid-DNA extraction) supplemented with KAN (50 mg/L), RIF (50 mg/L), and TET (30 mg/L). Background controls of all digestates and the recipients (100 μL of 10^-2^ dilution) were plated in duplicate on the corresponding selective media. Numbers of recipient cells were determined by applying three replicate 20 μL drops per each serial dilution of all mating mixes on PCA with KAN (50 mg/L) and RIF (50 mg/L). Transfer frequencies were calculated based on the following formula:

Transfer frequency: CFU mL^-1^ of transconjugants/CFU mL^-1^ of recipients.

### EXTRACTION OF PLASMID-DNA FROM TRANSCONJUGANTS AND TRANSFORMANTS

Plasmid-DNA was extracted from transconjugants of *P. putida* KT2442 *gfp*^+^ and *E. coli* CV601 *gfp*^+^ and transformed *E. coli* DH5α using the NucleoSpin® Plasmid kit (Macherey-Nagel, Düren, Germany) according to the manufacturer’s instructions for isolation of low copy plasmids.

### CONFIRMATION OF TRANSCONJUGANTS

Transconjugants were confirmed by their green fluorescence and by DNA-based methods. For the latter purpose, plasmid-DNA extracts of putative transconjugants (still containing genomic DNA) were tested either for the presence of *gfp* via PCR as described by Andersen et al. (1998) or by comparing the resulting BOX-PCR fingerprints to those of the corresponding recipient strain (Versalovic et al., 1994).

### CHARACTERIZATION OF PLASMIDS BY PCR AND SOUTHERN BLOT HYBRIDIZATION

Sequences specific for IncP-1, IncN, IncW, IncP-7, IncP-9, LowGC, and IncQ plasmids, the integrase gene *intI1*, TET-resistance gene *tet*(A) and regions flanking class 1 integron gene cassettes were detected in plasmid extracts using primer sets and PCR assays previously described (**Table 2**). Detection of IncP-1 plasmids (*trfA*) was performed using the primer systems targeting subgroups α, β, ε, δ, and γ as established by Bahl et al. (2009) and two novel primer pairs (**Table 2**) targeting the subgroup of IncP-1ζ plasmids (Norberg et al., 2011) and the IncP-1γ plasmid pKS208 (Heuer et al., 2002; referred to as γ-like in **Table 2**), which was not amplified with the primer system mentioned above. The primers were designed following the same concept as Bahl et al. (2009) based on the *trfA* sequences available for pKS208 (Sen et al., 2013) and the IncP-1ζ plasmids pMCBF1 and pMCBF6 (Norberg et al., 2011). Primer sequences and PCR conditions are given in **Table 2**. The primers described by Sandvang et al. (1997) bind to conserved sequences flanking the variable gene cassette region of class 1 integrons and thus are suitable for amplification of the gene cassette array present.

**Table 2:** Primer systems used in this study for characterization of captured plasmids.

Target gene	Primers	Sequence [5′–3′]	Amplicon size [bp]	Annealing temp.	Reference
IncN (*rep*)	IncN-rep-1 IncN-rep-2	agttcaccacctactcgctccg caagttcttctgttgggattccg	165	55°C	Götz et al. (1996)
IncP-1 (*trfA*)	trfA 733 f (α, β, ε) trfA 1013 r trfA g-F (γ) trfA g-R trfAg-208f (γ-l.) trfAg-208r trfA d-F (δ) trfA d-r trfA z-f (ζ) trfA z-r	ttcacsttctacgagmtktgccaggac gwcagcttgcggtacttctccc ttcactttttacgagctttgcagcgac gtcagctcgcggtacttctccca ttcaccttctacgaactgtgtaat gtcaaggcccgatacttctccca ttcacgttctacgagctttgcacagac gacagctcgcggtacttttccca ttcactttctacgaaatctgcaaagac gatagcttccgatacttttccca	281	60°C	Bahl et al. (2009); this paper
IncP-7 (*rep*)	P7repA (reverse) P7repB (forward)	ccctatctcacgatgctgta gcacaaacggtcgtcag	524	54°C	Izmalkova et al. (2005)
IncP-9 (*oriV*-*rep*)	IncP-9 ori 69f IncP-9 rep 679r	gagggtttggagatcat(at)aga ggtctgtatccagtt(ag)tgctt	∼610	53°C	Dealtry et al. (2014)
IncQ (*oriV*)	IncQ-oriV-1 IncQ-oriV-2	ctcccgtactaactgtcacg atcgaccgagacaggccctgc	436	57°C	Götz et al. (1996)
IncW (*oriV*)	IncW-oriV-1 IncW-oriV-2	gacccggaaaaccaaaaata gtgagggtgagggtgctatc	1140	57°C	Götz et al. (1996)
LowGC (*rep*)	V216repF V216repR	aattgaccgatttagttgtgacctgc tgatttgytttggagatac	912	56°C	Heuer et al. (2009)
*intI1*	intI1F intI1R	cctcccgcacgatgatc tccacgcatcgtcaggc	280	55°C	Kraft et al. (1986)
*tet*(A)	TetA-L TetA-R	ggcggtcttcttcatcatgc cggcaggcagagcaagtaga	502	64°C	Lanz et al. (2003)
*Integron gene cassettes*	5′-CS: intIF 3′-CS: intIB	ggcatccaagcagcaagc aagcagacttgacctgat	variable	55°C	Sandvang et al. (1997)

To increase specificity of detection, Southern blotting and hybridization were performed as described previously (Binh et al., 2008). Briefly, PCR products were run on agarose gels and subsequently blotted to a Hybond-N membrane (GE Healthcare, Buckinghamshire, UK) according to Sambrook et al. (1989). The membranes were hybridized with digoxigenin (DIG) labeled probes generated from PCR amplicons obtained with reference plasmids (*intI1*: pKJK5, *tet*(A): pKJK5, IncN: RN3, IncP-1α: RP4, IncP-1β: R751, IncP-1γ: pQKH54, IncP-1γ-like: pKS208, IncP-1δ: pEST4011, IncP-1ε: pKJK5, IncP-1ζ: pMCBF1, IncP-7: pCAR1, IncP-9: mixed probe (described by Dealtry et al., 2014), IncQ: RSF1010, IncW: R388, LowGC: pHHV216). The PCR-products of the integron gene cassettes were hybridized with a probe specific for the aminoglycoside resistance gene *aadA1* (Binh et al., 2009).

### SCREENING OF INCP-1ε PLASMIDS BY REAL-TIME PCR

Integrase genes of class 1 (*intI1*) and class 2 (*intI2*) integrons, ARG *sul1*, *qacEΔ1* (conferring resistance toward quaternary ammonium compounds) and sequences specific for plasmids of the IncP-1ε subgroup (*trfA*) were detected by real-time PCR 5′-nuclease assays in a CFX96 real-time PCR detection system (Bio-Rad, Hercules, CA, USA) as previously described (Heuer and Smalla, 2007; Barraud et al., 2010; Heuer et al., 2012; Jechalke et al., 2014a).

### TRANSFORMATION OF CAPTURED PLASMIDS INTO *E. coli* DH5α

Prior to further characterization, 16 exogenously isolated IncP-1ε plasmids were selected, based on the sizes of integron gene cassette amplicons, and transferred by transformation into *E. coli* DH5α via electroporation. For this purpose electrocompetent cells of *E. coli* DH5α were prepared using the protocol described by Choi et al. (2006). Fifty μL of freshly prepared electrocompetent cells were mixed on ice with 5–10 μL of plasmid-DNA extracts and transferred into electroporation cuvettes (2 mm electrode gap, Bulldog Bio, Portsmouth, NH, USA). Immediately after applying a pulse of 2,500 V, a volume of 950 μL Super Optimal Broth medium containing 20 mM glucose (SOC medium, provided at RT) was added and transformation mixtures were transferred into 1.5 mL Eppendorf tubes and incubated shaking at 300 rpm and 37°C for 1.5 h. To select for TET-resistant transformants, 200 μL of each transformation mix as well as pelleted (2 min, max. speed, mini Spin, Eppendorf, Hamburg, Germany) remaining cells resuspended in 100 μL SOC medium were streaked on LB agar supplemented with TET (15 mg/L). Plates were incubated at 37°C for 24 h and checked for growth of transformants.

### DETERMINATION OF ANTIBIOTIC RESISTANCE PATTERNS OF CAPTURED PLASMIDS

Single colonies of transformants were suspended in 900 μL sterile 0.85% NaCl solution by vortexing. 200 μL of the suspensions were streaked in triplicates onto Mueller-Hinton agar using sterile glass spatula. Paper disks containing different antibiotics of defined amounts (Becton, Dickinson and Company, Heidelberg, Germany) were placed on the inoculated plates. The plates were incubated at 37°C for 24 h and inhibition zones (IZ) around antibiotic paper disks were measured and compared to those of the plasmid-free recipient strain *E. coli* DH5α. Resistance was tested against the following antibiotics and concentrations: doxycycline (DOX) 30 μg, ampicillin 25 μg, trimethoprim (TMP) 5 μg, enrofloxacin 5 μg, SDZ 25 μg, chloramphenicol (CM) 30 μg, streptomycin (SM) 10 μg, cefotaxim 5 μg. Strains were considered resistant toward an antibiotic if the IZ around the respective paper disk was <8 mm, moderately susceptible if the IZ was 8–18 mm, and susceptible if the IZ was >18 mm.

### DETERMINATION OF RESTRICTION PATTERNS OF PLASMIDS

Plasmid-DNA was extracted from the *E. coli* DH5α transformants as described above. To evaluate the plasmid diversity, the plasmid-DNA was digested using NotI (Thermo Fisher Scientific, Waltham, MA, USA) and separated subsequently on a 1% agarose gel. The resulting digestion patterns were compared and grouped according to the number of fragments and corresponding fragment sizes.

### PLASMID REPLICON TYPING

A total of 10 transconjugants (five *P. putida* KT2442 *gfp*^+^ transconjugants from BGP1, BGP2, BGP3, BGP4, BGP5, and five *E. coli* CV601*gfp*^+^ transconjugants from BGP1) for which the plasmids captured could not be identified by PCR and Southern blot hybridization as described above, were analyzed applying the PBRT kit for PCR-based plasmid replicon typing (Diatheva, Fano, Italy). This kit is suitable for the detection of the plasmid incompatibility groups HI1, HI2, I1, I2, X1, X2, L/M, N, FIA, FIB, FIC, FII, FIIS, FIIK, W, Y, P, A/C, T, K, U, R, B/O, HIB-M, and FIB-M (representative for major plasmid incompatibility groups for resistance plasmids of *Enterobacteriaceae)*.

## RESULTS

### TRANSFERABLE ANTIBIOTIC RESISTANCE PLASMIDS CAPTURED FROM BIOGAS DIGESTATES

**FIGURE 1:**
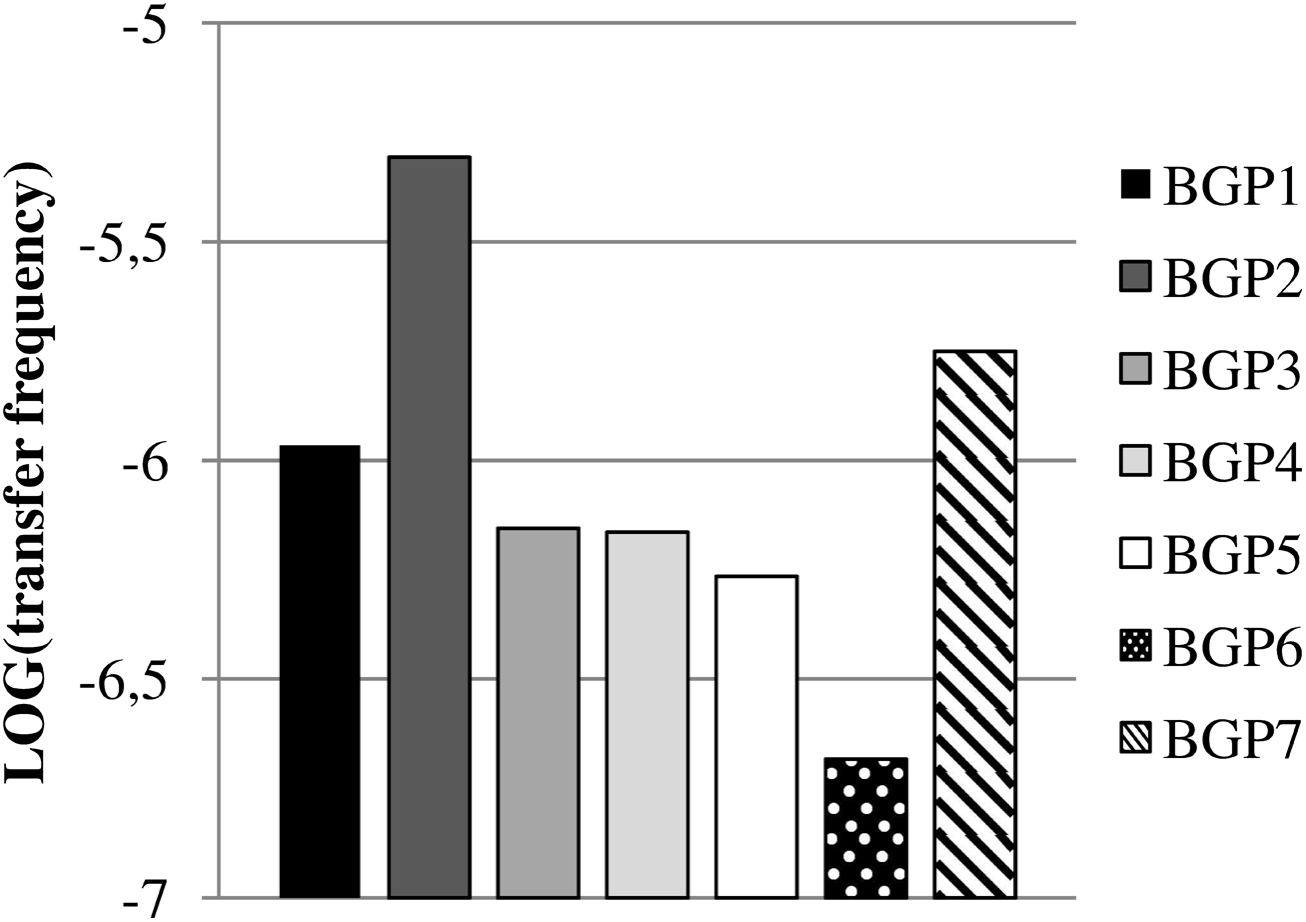
**Transfer frequencies of tetracycline-resistance from digestates of biogas plants (BGPs) into *Pseudomonas putida* KT2442 *gfp*^+^ via exogenous plasmid isolation.** Values are shown as logarithms of mean values (*n* = 2).

Exogenous plasmid isolations from the digestates were performed in order to capture transferable plasmids conferring resistance toward TET or SDZ into *P. putida* KT2442 *gfp*^+^ and *E. coli* CV601 *gfp*^+^ recipients, respectively. TET resistance was captured successfully from all digestates into *P. putida* KT2442 *gfp*^+^ at log transfer frequencies in the range of -5.3 to -6.7 (**Figure 1**). In contrast, transferable SDZ resistance plasmids could be captured into *E. coli* CV601 *gfp*^+^ only from the digestates of BGP1 (log transfer frequency of -5.6), because a background growth of indigenous bacteria resistant to SDZ, KAN, and RIF prevented the selection of transconjugants for samples derived from the other BGPs. Indeed, DNA extracted from the respective putative transconjugants obtained from BGP2, BGP3, BGP4, BGP5, BGP6, and BGP7 displayed BOX-patterns very different from the recipient, indicating background growth of multidrug-resistant bacteria.

### INCP-1ε PLASMIDS FREQUENTLY DETECTED AMONG TRANSCONJUGANTS FROM DIGESTATES

A total of 101 transconjugants were confirmed based on their growth on selective media and additionally by *gfp-* and/or BOX-PCR. PCR-Southern blot hybridization for sequences specific for several plasmid incompatibility groups of plasmids (**Table 3**) revealed that 40 plasmids could be assigned to the IncP-1 group. Further characterization by real-time PCR with primers specific for IncP-1ε showed that all IncP-1 plasmids belonged to the IncP-1ε plasmid group. IncP-1ε plasmids were captured from digestates of BGP2, BGP5, BGP6, and BGP7 using *P. putida* KT2442 *gfp*^+^ and from BGP1 using *E. coli* CV601 *gfp*^+^ as recipients. The plasmids contained in the remaining transconjugants could not be assigned to any of the plasmid groups tested, and their diversity was not further analyzed here.

**Table 3:** Detection of plasmids of different incompatibility groups and integrase genes specific for integrons of class 1 (*intI1*) in plasmid-DNA extracted from *Pseudomonas putida* KT2242 *gfp^+^* (Ps) and *Escherichia coli* CV601 *gfp^+^* (E) transconjugants via PCR and real-time PCR (BGP, biogas plant).

BGP	Recipient	IncN	IncP-1	IncP-7	IncP-9	IncQ	IncW	LowGC	*intI1*	Total number tested
1	Ps	–	–	–	–	–	–	–	–	10
2	Ps	–	8/9	–	–	–	–	–	8/9	9
3	Ps	–	–	–	–	–	–	–	–	23
4	Ps	–	–	–	–	–	–	–	–	10
5	Ps	–	4/9	–	–	–	–	–	4/9	9
6	Ps	–	10/10	–	–	–	–	–	10/10	10
7	Ps	–	9/9	–	–	–	–	–	9/9	9
1	E	–	9/21	–	–	–	–	–	9/21	21

All IncP-1ε plasmids were tested positive by PCR-Southern blot hybridization for integrase genes of class 1 integrons (*intI1*), the TET-resistance gene *tet*(A) and the sulfonamide resistance gene *sul1.* In addition, all IncP-1ε plasmids contained integron gene cassette amplicons with sizes varying from 1,000 to 2,300 bp (**Tables 3** and (**Tables 3 and 4**) and gene cassette PCR products of 15 IncP-1ε plasmids were tested positive for *aadA1* by Southern blot hybridization (**Table 4**). The gene cassette amplicons of the remaining 25 IncP-1ε plasmids weakly hybridized with the *aadA1* probe, suggesting the presence of other *aadA* variants.

**Table 4:** Detection of integron gene cassette amplicons, antibiotic resistance genes *tet*(A) and *sul1* and genes conferring resistance against quaternary ammonium compounds (*qacEΔ1*) in IncP-1ε plasmid-DNA extracted from *P. putida* KT2242 *gfp*^+^ (Ps) and *E. coli* CV601 *gfp^+^* (E) transconjugants via PCR, Southern blot hybridization and real-time PCR [(+) = weak hybridization signal after long exposure time; BGP, biogas plant].

BGP	Recipient	Gene cassette amplicon sizes [bp] (number positive/tested)	*qacEΔ1*	*tet*(A)	*sul1*	*aadA1*
2	Ps	1000 (6/8) 1600 (1/8) 1700 + 2300 (1/8)	+ + +	+ + +	+ + +	+ (+) +
5	Ps	1000 (1/4) 1700 (3/4)	+ +	+ +	+ +	+ +
6	Ps	1500 (4/10) 2200 (6/10)	+ +	+ +	+ +	(+) (+)
7	Ps	1700 + 2300 (1/9) 2200 (8/9)	+ +	+ +	+ +	+ (+)
1	E	2000 (2/9) 2000 + 1000 (1/9) 2200 (6/9)	+ + +	+ + +	+ + +	+ + (+)

### INCP-1ε PLASMIDS CONFERRED MULTIPLE ANTIBIOTIC RESISTANCES

Based on the varying size of the gene cassette amplicons, a total of 16 IncP-1ε plasmids (representative for 37 of the 40 IncP-1ε plasmids) was chosen for further analysis. For this purpose, they were transferred into *E. coli* DH5α by electroporation to make possible a comparative characterization in an identical genetic background. Antibiotic resistances conferred by plasmids determined by disk diffusion tests revealed six different antibiotic resistance patterns (**Table 5**) compared to the plasmid-free *E. coli* DH5α which was susceptible toward all antibiotics tested. Each of the eight plasmids harboring a gene cassette amplicon of 2,200 bp (Ps128, Ps152, Ps154, Ps156, E9, E10, E12, and E17) displayed resistance toward SDZ, TET, TMP, and moderate resistance toward DOX and SM (pattern #1). However, the same resistances were also conferred by the plasmids containing an integron gene cassette amplicon of 1,600 bp (Ps28) and 1,500 bp (Ps151). Both plasmids (Ps29 and Ps134) in which integron gene cassette amplicons of both 2,300 and 1,700 bp were detected showed the resistance toward SDZ, CM, SM, TET, and moderate resistance toward DOX (pattern #2). The size of the integron gene cassette amplicons of remaining plasmids was either 1,000 or 1,700 bp and displayed unique resistance patterns (pattern #3–6). Thus, in some cases different resistance patterns were found to be related to the same size of gene cassette amplicons from class 1 integrons. Plasmids conferring resistances to the same antibiotics were captured from different BGPs. Although only plasmids displaying resistance pattern #1 were captured from BGP1, pattern #1 was also found in plasmids originating from BGP6 and BGP7. Furthermore, the different representative plasmids of resistance pattern #2 were captured from digestates of different BGPs.

**Table 5:** Plasmid specific sequences, resistance genes and sizes of gene cassette amplicons detected via PCR and real-time PCR in DNA of *E. coli* DH5α transformants (TF) of plasmids captured from digestates of different biogas plants (BGPs) and corresponding antibiotic resistance patterns as determined by antibiograms (Ps = plasmid originally captured in *P. putida* KT2442 *gfp*^+^; E = plasmid originally captured in *E. coli* CV601*gfp*^+^; *determined by growth on media containing 15 mg tetracycline (TET) per liter; rp, resistance pattern; CM, chloramphenicol; DOX, doxycycline; SDZ, sulfadiazine; SM, streptomycin; TMP, trimethoprim).

#TF	BGP	Detected markers	Gene cassette size [bp]	Resistance	Moderate resistance	# rp
Ps26	2	IncP-1ε (*trfA*), *intI1*, *qacEΔ1*, *sul1*, *tet*(A), *aadA1*	1000	SDZ, TET*	DOX	3
Ps28	2	IncP-1ε (*trfA*), *intI1*, *qacEΔ1*, *sul1*, *tet*(A)	1600	SDZ, TET*, TMP	DOX, SM	1
Ps29	2	IncP-1ε (*trfA*), *intI1*, *qacEΔ1*, *sul1*, *tet*(A), *aadA1*	2300 + 1700	SDZ, CM, SM, TET*	DOX	2
Ps32	2	IncP-1ε (*trfA*), *intI1*, *qacEΔ1*, *sul1*, *tet*(A), *aadA1*	1000	SDZ, SM, TET*	DOX	4
Ps101	5	IncP-1ε (*trfA*), *intI1*, *qacEΔ1*, *sul1*, *tet*(A), *aadA1*	1700	SDZ, SM, TET*	DOX, CM	5
Ps110	5	IncP-1ε (*trfA*), *intI1*, *qacEΔ1*, *sul1*, *tet*(A), *aadA1*	1000	SDZ, TET*	DOX, SM	6
Ps151	6	IncP-1ε (*trfA*), *intI1*, *qacEΔ1*, *sul1*, *tet*(A)	1500	SDZ, TET*, TMP	DOX, SM	1
Ps152	6	IncP-1ε (*trfA*), *intI1*, *qacEΔ1*, *sul1*, *tet*(A)	2200	SDZ, TET*, TMP	DOX, SM	1
Ps154	6	IncP-1ε (*trfA*), *intI1*, *qacEΔ1*, *sul1*, *tet*(A)	2200	SDZ, TET*, TMP	DOX, SM	1
Ps156	6	IncP-1ε (*trfA*), *intI1*, *qacEΔ1*, *sul1*, *tet*(A)	2200	SDZ, TET*, TMP	DOX, SM	1
Ps128	7	IncP-1ε (*trfA*), *intI1*, *qacEΔ1*, *sul1*, *tet*(A)	2200	SDZ, TET*, TMP	DOX, SM	1
Ps134	7	IncP-1ε (*trfA*), *intI1*, *qacEΔ1*, *sul1*, *tet*(A), *aadA1*	2300 + 1700	SDZ, CM, SM, TET*	DOX	2
E9	1	IncP-1ε (*trfA*), *intI1*, *qacEΔ1*, *sul1*, *tet*(A)	2200	SDZ, TET*, TMP	DOX, SM	1
E10	1	IncP-1ε (*trfA*), *intI1*, *qacEΔ1*, *sul1*, *tet*(A)	2200	SDZ, TET*, TMP	DOX, SM	1
E12	1	IncP-1ε (*trfA*), *intI1*, *qacEΔ1*, *sul1*, *tet*(A)	2200	SDZ, TET*, TMP	DOX, SM	1
E17	1	IncP-1ε (*trfA*), *intI1*, *qacEΔ1*, *sul1*, *tet*(A)	2200	SDZ, TET*, TMP	DOX, SM	1

Comparing the detected marker genes and the respective resistance patterns observed (**Table 5**), all transformants negative for *aadA1* displayed moderate resistance toward streptomycin and were resistant toward TMP. Vice versa, none of the plasmids that harbored *aadA1* within their integron gene cassettes conferred resistance toward TMP. Also it is worth noting that in contrast to all other transformants tested positive for *aadA1*, the transformant Ps26 displayed no resistance toward SM.

**FIGURE 2:**
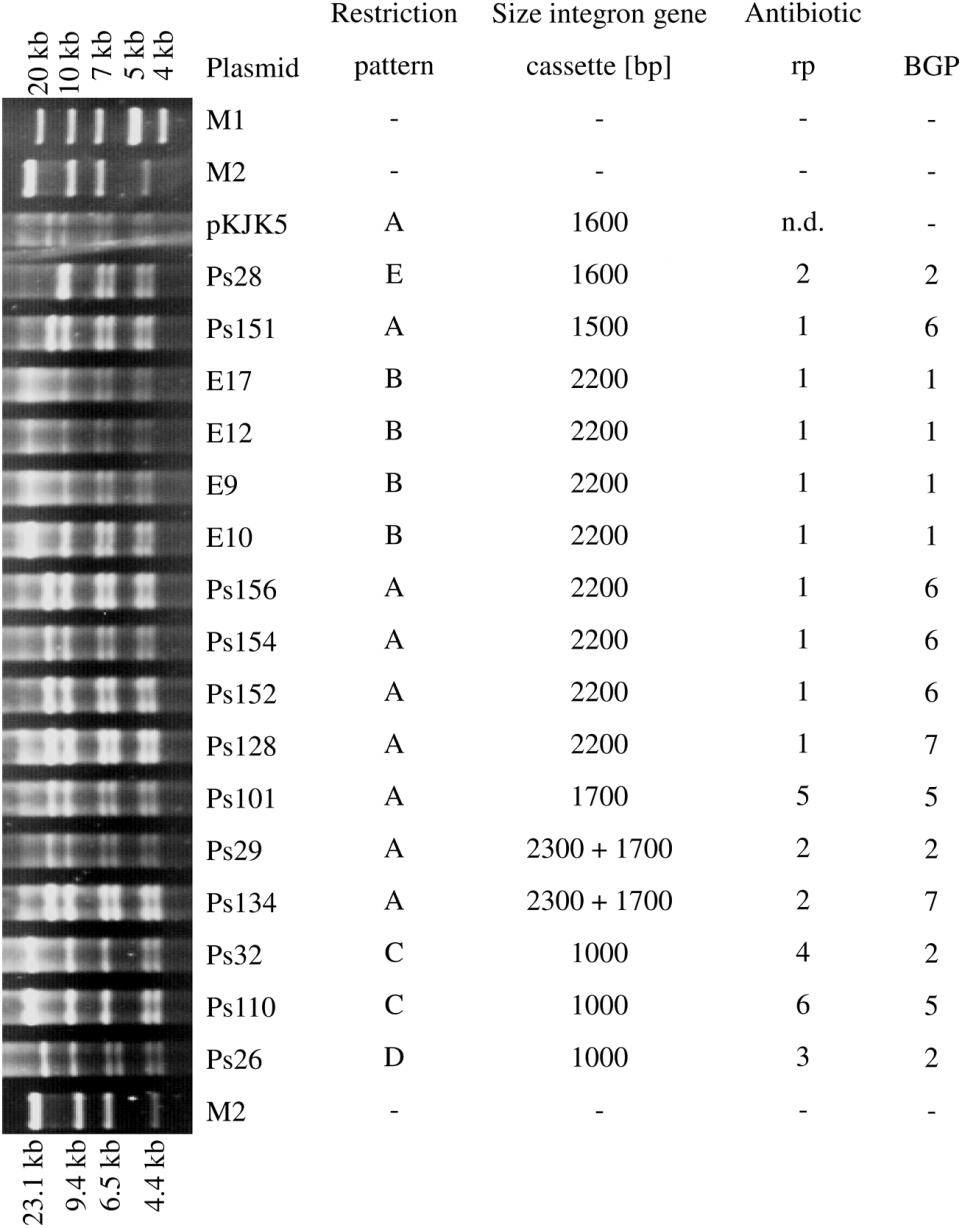
**NotI restriction patterns of transformed plasmids, corresponding sizes of integron gene cassettes, antibiotic resistance patterns (rp) and biogas plant (BGP) they were derived from (M1 = GeneRuler^TM^ 1 kb Plus DNA ladder, Thermo Fisher Scientific, Waltham, MA, USA; M2 = DNA Molecular Weight Marker II, DIG-labeled, Roche Diagnostics, Rotkreuz, Switzerland)**.

The restriction analysis of the IncP-1ε plasmids transferred into *E. coli* DH5α revealed five different patterns (**Figure 2**). Eight plasmids, originating from BGP2, BGP5, BGP6, and BGP7, shared restriction pattern A which was also observed for the IncP-1ε type plasmid pKJK5. These plasmids were all originally captured into *P. putida* KT2442 *gfp*^+^. Surprisingly, plasmids of the restriction pattern A contained gene cassette amplicons of varying sizes and displayed three different antibiotic resistance patterns (#1, #2, and #5). The four plasmids captured into *E. coli* CV601 *gfp*^+^ all displayed restriction pattern B, harbored an integron gene cassette amplicon of 2,200 bp, and displayed resistance pattern #1. Although sharing the same restriction pattern C and containing integron gene cassette amplicons of the same size, the plasmids Ps32 and Ps110 represented different antibiotic resistance profiles. The plasmids Ps26 and Ps28 displayed unique restriction patterns among this set of plasmids. Both displayed antibiotic resistance pattern #2 which was also determined for one plasmid showing restriction pattern A (Ps29). Considering these results, it was not possible to correlate the restriction patterns of the IncP-1ε plasmids to the different resistance profiles observed, sizes of integron gene cassette amplicons, or to their different origins.

### PLASMID REPLICON TYPING OF UNIDENTIFIED PLASMIDS

None of the unassigned plasmids from the 10 transconjugants could be affiliated to any of the plasmid groups (HI1, HI2, I1, I2, X1, X2, L/M, N, FIA, FIB, FIC, FII, FIIS, FIIK, W, Y, P, A/C, T, K, U, R, B/O, HIB-M, and FIB-M) surveyed by the PBRT kit for PCR-based plasmid replicon typing.

## DISCUSSION

In previous studies, the effect of anaerobic fermentation has been tested for its mitigation potential toward pathogens and multidrug resistant bacteria introduced into the fermenters with substrates, such as manures (Iwasaki et al., 2011; Beneragama et al., 2013; Resende et al., 2014). It has been shown that bacteria belonging to both categories may survive this procedure if the fermentation is performed under mesophilic temperatures. Digestates of mesophilic BGPs represent, therefore, a potential reservoir of pathogens and bacteria displaying antibiotic resistances. But so far there is a lack of knowledge concerning the transferability of ARGs and MGEs harbored in bacteria resident in digestates to other bacterial hosts. This is the first report on conjugative plasmids captured in *P. putida* KT2442 *gfp*^+^ and *E. coli* CV601 *gfp*^+^ recipients from digestates of several different mesophilic full-scale BGPs, fermenting manures as co-substrate, based on resistances conferred toward two classes of antibiotics (i.e., TETs and sulfonamides) most commonly applied in livestock in Germany (Federal Office of Consumer Protection and Food Safety, Germany, 2014^2^).

Manures have been shown to represent a reservoir of transferable antibiotic resistance plasmids such as IncN, IncP-1, IncW, LowGC, and IncQ plasmids (Smalla et al., 2000; Heuer et al., 2002, 2009; Binh et al., 2008). Except for LowGC, all these mentioned incompatibility groups of plasmids display a broad host range. This means they are able to self-transfer (IncN, IncP-1, IncW) or to be mobilized (IncQ) to, replicate in and be stably maintained in phylogenetically distant bacteria. It was assumed that broad host range plasmids play an important role in interspecies gene transfer and adaptation of bacterial communities to environmental stresses. Thus, if these plasmids provide a fitness advantage to their hosts, there is a chance for their persistence once transferred into a bacterial host associated with environments that might differ from those of the original plasmid host.

In order to avoid the limitations of solely DNA-based methods (e.g., detection of relicts of dead bacteria) or cultivation-dependent methods (which only allow to analyze culturable bacteria) we performed exogenous plasmid isolation (Bale et al., 1988) in this study to capture transferable plasmids conferring resistance to either TETs or SDZ from the digestates. Although the choice of recipient strains is crucial for the successful capture of different plasmids (due to the need of maintenance and expression in the new host), this is the only method allowing for the unambiguous detection of MGE-associated ARGs that is not limited by the culturability of the original bacterial host.

In this study, antibiotic resistance plasmids were captured successfully from all digestates when using TET as selective agent, while a high background growth occurred when using SDZ to select for transconjugants. This background growth appeared due to the presence of bacteria displaying an antibiotic resistance phenotype toward SDZ, KAN, and RIF, which seemed to be highly prevalent among indigenous digestate bacteria. Thus, transferable plasmids conferring SDZ resistance could be captured in *E. coli* CV601 *gfp*^+^ recipients only from digestates of BGP1. The growth of multidrug resistant bacteria originating from BGP digestates is in accordance with a study by Beneragama et al. (2013) who at lab-scale compared the survival of multidrug resistant bacteria residing in dairy manure during mesophilic and thermophilic anaerobic digestion. They found that after treatment under mesophilic conditions (similar to those prevailing in the BGPs sampled for our study) those bacteria were still viable, although largely reduced in number, while thermophilic treatment eliminated them. As we did not have problems with background growth on TET-KAN-RIF selective plates, it seems that the association of TET resistance with KAN and RIF resistance in the indigenous BGP bacteria was less common.

A substantial proportion (40/101) of the isolated plasmids captured during this study could be assigned to the subgroup of IncP-1ε plasmids (**Table 3**). Those plasmids were originating from digestates of different BGPs. At the same time, plasmids of this group were detected only at a low abundance in the respective TC-DNA of the analyzed digestates by PCR-Southern blot hybridization and qPCR, and were in some cases even below the detection limit (Wolters et al., data not shown). These data indicate a clear advantage of the exogenous plasmid isolation technique to isolate plasmids that occur at low abundance but transfer efficiently. These plasmids would likely not be captured by metagenomic approaches. This observation confirmed the already known outstanding transfer efficiency of IncP-1 plasmids (Pukall et al., 1996; Shintani et al., 2010). The host range of this plasmid group is known to be broad, enclosing Gram-negative bacteria (*Alpha*-, *Beta*-, and *Gammaproteobacteria*), and they have been shown to be also transferable to *Arthrobacter* sp. (Gram-positive) in which, however, their replication was not analyzed (Musovic et al., 2006; Shintani et al., 2010, 2014; Klümper et al., 2014). Originally, IncP-1 plasmids were discovered in clinical isolates (Datta et al., 1971). Nowadays, plasmids of this group were reported from various environments such as soil, manure, sludge, water, and river sediments (Top et al., 1994; Götz et al., 1996; Haines et al., 2006; Smalla et al., 2006; Heuer and Smalla, 2012). Additionally, they have been shown to be transferable to bacteria associated with the rhizosphere of different plant species (Musovic et al., 2006; Mølbak et al., 2007). Moreover, IncP-1 plasmids were frequently captured by exogenous plasmid isolation from different environments such as manures, manured soils, and sludges before (Top et al., 1994; Heuer et al., 2002, 2012; Binh et al., 2008). The ability of IncP-1 plasmids to efficiently transfer in surface matings likely contributes to their frequent capturing by means of the exogenous plasmid isolation.

The co-occurrence of the sulfonamide resistance gene *sul1*, the integrase gene of class 1 integrons (*intI1*), gene cassette amplicons of different size (**Table 4**) and of *qacEΔ1*, was already shown by Heuer et al. (2012) and Jechalke et al. (2014a) in IncP-1ε plasmids exogenously isolated from manure and arable soil, using SDZ as a selection marker. Those IncP-1ε plasmids contained similar components as the plasmids reported here (*intI1*, *sul1,* and gene cassette amplicons that in many cases hybridized to an *aadA* specific probe). The different sizes of gene cassette amplicons observed in the study by Heuer et al. (2012) were in the same range as the sizes determined for the amplicons of our IncP-1ε plasmids that were isolated based on the antibiotic resistance conferred toward TET (in case of *P. putida* KT2442 *gfp*^+^ transconjugants) or SDZ (*E. coli* CV601 *gfp*^+^ transconjugants). The finding that IncP-1ε plasmids were captured from manures using the same methods as in our study, and that they carried clinical class 1 integrons harboring gene cassettes of sizes similar to those observed for the plasmids isolated from BGP digestates is not too surprising as manure was used in all BGPs. Thus, it is most likely that these plasmids originated from manure bacteria, and this might be a hint for the survival of these bacteria during mesophilic processing in full-scale BGPs. Interestingly, none of both completely sequenced IncP-1ε plasmids exogenously isolated from a Norwegian soil by Sen et al. (2011), based on the mercury resistance conferred, harbored integrons. The presence of *qacEΔ1* in all 16 IncP-1ε plasmids captured from BGPs is in accordance with its detection in TC-DNA derived from BGP digestates by Jechalke et al. (2014a). Due to the frequent application of disinfectants containing quaternary ammonium compounds in pig producing systems (Preller et al., 1995) it is possible that a selection for bacteria carrying plasmids with clinical class 1 integrons might have occurred in the manures before they were fermented in the BGPs sampled in our study.

The ARG *tet*(A) which confers resistance toward TET by an eﬄux mechanism was detected in all IncP-1ε plasmids of the present study, also in those plasmids that were captured based on the SDZ-resistance conferred. Similarly, the three completely sequenced IncP-1ε plasmids isolated from arable field soil (pHH3414, pHH128) and pig manure (pKS77) reported by Heuer et al. (2012), and the completely sequenced IncP-1ε archetype of this plasmid subgroup, pKJK5, which was originally isolated from manured soil, were shown to harbor this gene as well (Bahl et al., 2007). The high proportion of plasmids carrying *sul1* and *tet*(A) is likely a consequence of the antibiotic selection pressure posed by the extensive application of TETs and sulfonamides in livestock.

In addition, within the integron gene cassette amplicons of six plasmids transferred into *E. coli* DH5α transformants the aminoglycoside resistance gene *aadA1* could be detected. Very weak hybridization signals were also obtained for further transformants using the probe specific for *aadA1*. This might be a hint for the presence of other *aadA* gene variants that share sequence similarity to *aadA1*. *aadA* gene cassettes are known to be frequently inserted in class 1 integrons. For example, a study by Binh et al. (2009) revealed a high abundance of several different *aadA* genes harbored in integrons of class 1 detected in samples derived from pig manures and manured soils.

The observation made in this study that all IncP-1ε plasmids characterized in more detail harbored the genes *tet*(A), *sul1*, *qacEΔ1,* and *intI1* is especially interesting with regard to the results of Forsberg et al. (2012). They compared sequencing data from pooled genomic DNA of multidrug-resistant isolates from soil samples with sequences of known human pathogenic isolates and reported four fragments obtained from multidrug-resistant soil isolates, which shared >99% nucleotide identity with sequences of human pathogens. Within these fragments they found the same genes [*intI1*, *qacEΔ1*, *sul1,* and *tet*(A)] which were also detected in the present study on all 16 IncP-1ε plasmids from BGPs. Moreover, the majority of integron gene cassettes of human pathogenic isolates that were used for comparison in their study contained aminoglycoside resistance genes such as *aadA* genes. The same holds true for at least six of our plasmids which contained *aadA1* localized on class 1 integrons. This suggests that IncP-1ε plasmids might be considered possible ARG vectors between environmental bacteria and human pathogens.

The observed correlation of antibiotic resistance patterns displayed by the plasmids reported here with the different sizes of integron gene cassette amplicons (**Table 5**) does once again reflect the importance of these genetic elements in the adaptation of bacterial hosts to antibiotic stress. The types of ARG cassettes inserted are likely determining the plasmid-conferred resistance profiles. Similarly, it was proposed by Heuer et al. (2012) that integron gene cassettes might be one of the main drivers of diversification of IncP-1ε plasmids.

It is noteworthy that the restriction pattern A which showed a high similarity to pKJK5 was the most frequent among the 16 plasmids (**Figure 2**). By sequence comparison of pKJK5 with four IncP-1ε plasmids originating from arable soil and pig manure, Heuer et al. (2012) found that the backbones of all four plasmids were 99.9% identical to pKJK5. IncP-1ε plasmids representing restriction pattern A were captured from digestates of four different BGPs (BGP2, BGP5, BGP6, and BGP7). Important to note was that plasmids sharing the same restriction patterns (namely A and C) conferred different antibiotic resistance patterns (**Figure 2**). This might also be a hint of the diversification of this plasmid group by integron gene cassette rearrangement, rather than whole-genome rearrangement.

Particularly striking is the correlation of resistance patterns conferred by the IncP-1ε plasmids reported here to the application patterns of antibiotics in livestock production. As already mentioned, especially TETs and sulfonamides, together with β-lactams account for the vast majority of antibiotics applied in animal husbandry, and also aminoglycosides and TMP are frequently administered (Federal Office of Consumer Protection and Food Safety, Germany, 2014^3^).

The remaining 61 plasmids, transferring resistance toward either TETs or sulfonamides, were not assigned to any incompatibility group tested. Even though not all known incompatibility groups of plasmids were included in our monitoring, the fact is striking that the major proportion (61/101; **Table 3**) of the plasmids captured could not be assigned to plasmid groups typically associated with manures. Also the plasmids of a small subset of 10 transconjugants (five *P. putida* KT2442 *gfp*^+^ transconjugants from BGP1, BGP2, BGP3, BGP4, BGP5, and five *E. coli* CV601*gfp*^+^ transconjugants from BGP1), which were analyzed by applying the PBRT kit for PCR-based plasmid replicon typing (Diatheva, Fano, Italy), could not be assigned. As most of the plasmids captured from manure could be assigned to known plasmid types (Binh et al., 2008) the plasmids might originate from bacteria of other co-substrates fermented. The so far undiscovered diversity of plasmids residing in digestates requires further investigation.

Concerning the potential dissemination of MGEs associated with ARGs via soil fertilization with digestates of mesophilic full-scale BGPs, it was clearly shown in our study that all analyzed digestates were sources of transferable antibiotic resistances. The observed transfer frequencies of the digestates (**Figure 1**) were comparable to those reported by Binh et al. (2008) who captured plasmids from pig manure by means of exogenous plasmid isolation based on either SDZ-resistance (at transfer frequencies ranging from 10^-4^ to 10^-8^) or TET-resistance (10^-4^ to 10^-8^) using *E. coli* CV601*gfp*^+^ as recipient. Due to the known broad host range of IncP-1ε plasmids (Musovic et al., 2006; Heuer et al., 2012) there might be a chance for all captured plasmids assigned to the group reported here to be transferred to soil- and plant-associated bacteria if the digestates are used as fertilizer. Since all analyzed plasmids conferred multiple antibiotic resistance and harbored integrons, they might contribute to the increasing problems caused by multi-resistant pathogens in clinical settings, nowadays threatening public health (Enright et al., 2002; Velayati et al., 2009; Davies and Davies, 2010).

## Conflict of Interest Statement

The authors declare that the research was conducted in the absence of any commercial or financial relationships that could be construed as a potential conflict of interest.

## Footnotes

1 http://www.biogas.org

2 http://www.bvl.bund.de

3 http://bvl.bund.de
